# AI and machine learning for soil analysis: an assessment of sustainable agricultural practices

**DOI:** 10.1186/s40643-023-00710-y

**Published:** 2023-12-07

**Authors:** Muhammad Awais, Syed Muhammad Zaigham Abbas Naqvi, Hao Zhang, Linze Li, Wei Zhang, Fuad A. Awwad, Emad A. A. Ismail, M. Ijaz Khan, Vijaya Raghavan, Jiandong Hu

**Affiliations:** 1 College of Mechanical and Electrical Engineering, Henan Agricultural University, Zhengzhou, 450002 China; 2 Henan International Joint Laboratory of Laser Technology in Agriculture Sciences, Zhengzhou, 450002 China; 3 Department of Quantitative Analysis, College of Business Administration, King Saud University, P.O. Box 71115, Riyadh 11587, Saudi Arabia; 4 Department of Mathematics and Statistics, Riphah International University, I-14, Islamabad, 44000 Pakistan; 5 Department of Mechanical Engineering, Lebanese American University, Kraytem, Beirut, 1102-2801 Lebanon; 6 Department of Bioresource Engineering, Faculty of Agriculture and Environmental Studies, McGill University, Sainte-Anne-de-Bellevue, QC H9X 3V9 Canada

**Keywords:** Intelligent agriculture, Agronomic forecasting, Soil texture, Water content analysis, Smarter agriculture 4.0

## Abstract

Sustainable agricultural practices help to manage and use natural resources efficiently. Due to global climate and geospatial land design, soil texture, soil–water content (SWC), and other parameters vary greatly; thus, real time, robust, and accurate soil analytical measurements are difficult to be developed. Conventional statistical analysis tools take longer to analyze and interpret data, which may have delayed a crucial decision. Therefore, this review paper is presented to develop the researcher’s insight toward robust, accurate, and quick soil analysis using artificial intelligence (AI), deep learning (DL), and machine learning (ML) platforms to attain robustness in SWC and soil texture analysis. Machine learning algorithms, such as random forests, support vector machines, and neural networks, can be employed to develop predictive models based on available soil data and auxiliary environmental variables. Geostatistical techniques, including kriging and co-kriging, help interpolate and extrapolate soil property values to unsampled locations, improving the spatial representation of the data set. The false positivity in SWC results and bugs in advanced detection techniques are also evaluated, which may lead to wrong agricultural practices. Moreover, the advantages of AI data processing over general statistical analysis for robust and noise-free results have also been discussed in light of smart irrigation technologies. Conclusively, the conventional statistical tools for SWCs and soil texture analysis are not enough to practice and manage ergonomic land management. The broader geospatial non-numeric data are more suitable for AI processing that may soon help soil scientists develop a global SWC database.

**Graphical Abstract:**

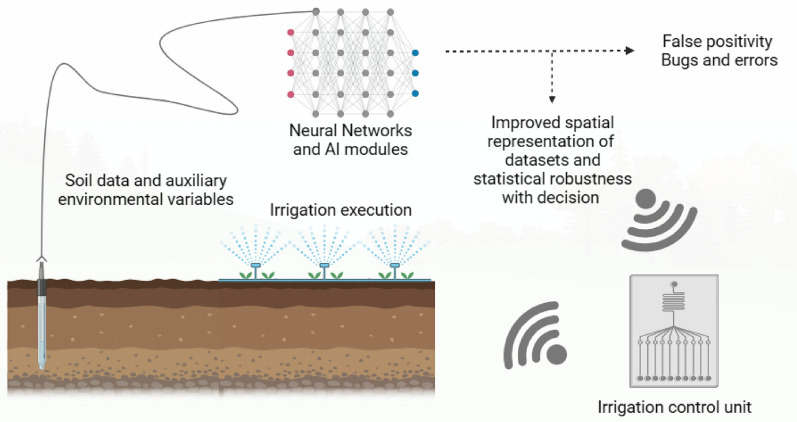

## Introduction

Organisms need water as a compulsory part of routine metabolic activities, and especially plants require a continual supply of soil–water to maintain their turgor and transport mechanisms (Huang et al. 2020). The predominant allocation of water within plants is dedicated to the hydrolysis process, which serves as a means of generating energy required for the sustenance of a diverse array of chemical reactions and physiological processes (Bhunia et al. 2023). Soil–water contents (SWCs) are often confused with soil–moisture contents (SMCs), which in general are different. SWCs account for a ratio between the volume of water present in a unit of soil volume (Hueso et al. 2012). It has been discovered that the rate of mineralization significantly affects the microbial contents and activity, which helps to regulate plant growth (Ma et al. 2022). Moreover, the soil porosity and SWC saturation in designated soil pores may hinder O_2_ diffusion, because the rate of O_2_ diffusion is about one hundred times less when compared with air.

Hence, the ideal soil–water contents SWCs are limited in their ability to sustain crop growth as a result of the impeded diffusion caused by saturated soil pores (Zhang et al. 2021). Similarly, when the saturation of soil pores decreases from an optimal level, it results in significant damage to the microbial flora residing within these pores. Consequently, this leads to a decline in nitrogen and carbon mineralization (Schlüter et al. 2098). Furthermore, when drought is induced in soil pores, the water films surrounding soil particles become thinner, and dryness prevails in the soil, which primes water channels in the soil pores to become disconnected (Dwevedi et al. 2017).

The optimal SWCs are closely associated with carbon allocation, plant growth, nutrient recycling, photosynthetic rate, and microbial activity. The regulation of these parameters has also invariably been linked with the physicochemical properties of water that are held in soil (Wang et al. 2023). There is a fuzzy concept that all of the water present in soil can be taken up by plants. However, this is not a true concept; holding the water against the gravitational pull is a soil art that most often depends on the type of soil. Another indicator of the soil's overall ability to store water is its porosity. Measuring soil–water content and potential is the initial step in doing soil research, since they are important to state quantities of soil (Heiskanen 1997; Vereecken et al. 2015). Therefore, this water-holding capacity against the gravitational pull feeds the crop during water scarcity posed by low precipitation.

Though, due to complicated laboratory protocols and high cost, it remains a mere challenge to analyze available water contents for plant growth (Zhao et al. 2015). Soil–water holding capacity (SWHC) is mainly affected by soil texture, which is further dependent upon pH, temperature, microbial community, precipitation, type of soil, and other relatable factors. For example, an elevated environmental temperature will lead to the thawing of permafrost, which leads to many fluctuations in soil properties finally nutrient availability to plants is compromised (Månsson et al. 2014). Changes in crop rotation and land use can make the soil better in ways, such as C/N ratio, bulk density, tillage (Beheshti et al. 2012), and soil–organic carbon distribution is influenced by general topography (Sun et al. 2015). Moreover, carbon stocks are influenced by precipitation. There are very few studies available on the subject that encapsulate multifactorial factors influencing soil’s porosity and texture (Jamil et al. 2016). For instance, nutrient movement, pore size, and soil structure are influenced by the soil physical properties. Soil fertility is improved by particle surface absorption of ions in clay (Wang et al. 2022). Hence, the comprehensive examination of environmental factors pertaining to soil texture, porosity, and water content availability necessitates a laborious endeavor (Dragone et al. 2020).

The electrical conductivity (EC) and SWC measurements with minimal damage to soil have been anticipated by scientists for many years (Masha et al. 2021). While from the previous literature, it has been found that these factors are greatly influenced by soil porosity, which in turn is compromised by fluctuating environmental influencers (Bittelli 2011). Besides, the effects of environmental influencing variables are a time-demanding and complex task (Ruszczak and Boguszewska-Mańkowska 2022) that has not been reviewed and has also not been experimentally evaluated in a single manuscript (Heiskanen 1997).

Due to the diversified data available, it is very difficult to establish and study the effects of all influencing factors on soil parameters (Zhao et al. 2020). This creates a huge hurdle to getting a complete insight into the influencing parameter and making further decisions for intelligent agricultural practices manually (Pastén-Zapata et al. 2014). The accurate outcomes and decisions may be facilitated by considering the processing time and prerequisites of both conventional and advanced statistical techniques (Clauser et al. 2022). However, on the other hand, the critical time to perform a management response may have surpassed. For instance, the SWCs have been recorded on the landmark, and it is time to irrigate. While conventional statistics play a significant role in this irrigation decision-making process, the relative analysis is consistently and flawlessly present (Blanco and Lal 2023).

Artificial intelligence (AI) has been found to process non-numerical data, such as images, videos, text, and voice data with greater perfection. Therefore, there is a need to align the geophysical influence data on soil quality with artificially intelligent systems to process the decision-making more robustly (Liu et al. 2014). The AI does not even require the data to be large enough to process and suggest a crop management practice. The AI tools have previously been found to be smart enough to remove the noise from SWC, EC, and DC data for soil parameters. The noise removal was found to give an accurate SWC measurement that is actually available for the crop to be taken (Ratshiedana et al. 2023). The current study explains a review of soil–water content data and its possible processing using AI tools. The false positivity in SWC results and bugs in advanced detection techniques are also evaluated, which may lead to wrong agricultural practices. This review first covers the soil–water relationships and advancements in measurement techniques for SWC and soil texture. Second, the initial efforts for the development of global SWC and soil texture databases using AI networks have been discussed. Furthermore, the conventional statistical and AI analysis platforms have been compared, and conclusions are drawn for future recommendations.

### The diverse SWCs inside the soil

The soil has the property of water anchorage, which may change with physicochemical texture and climate (Singh and Nair 2023). The water-holding capacity of soil can vary among different types of soil. However, the comprehensive depiction of soil–water is insufficient to elucidate the scientific principles governing water absorption by field crops (Adhikari et al. 2022). The soil–water can be attributed to different types, i.e., hygroscopic soil–water (HSW), gravitational soil–water (GSW), and capillary soil–water (CSW). These various varieties of soil–water are subject to distinct and variable forces that degrade the soil (Rayne and Aula 2020). The soil architecture is quite variable and is affected by various environmental factors that regulate the soil pore distribution; likewise, the soil–water distribution is affected (Jian et al. 2015). The HSW contents are held by soil–particle physical interactions in vapor form, which is more often hydrogen bonding. These contents are very unlikely to be strained by the crops for their growth due to strong soil binding (Wuddivira et al. 2012). After the precipitation, the GSW is rapidly increasing but is drained with more speed than any other type of soil–water due to its humongous gravitational pull. The gravitational forces drag GSW contents sharply to the larger pores deep down in the soil and often add to the water table (Fu et al. 2021). Due to shorter root lengths, the crops are also much less likely to use this GSW. As a general concept, GSW contents are temporarily available to the crops only before they are drained. Ideally, plants can easily access the soil–water when water contents are -33 bar; this only happens after all of the GSW drainage is completed and is termed field capacity (Leucci 2012). Water is usually considered the most important factor for crop growth, but actually, the soil–water content causes plants to wilt if all of the soil pores are filled with water (saturation). Total saturation hinders oxygen diffusion and halts the respiration of roots, which destroy the whole crop lot (Bhattarai et al. 2005). Therefore, the actual and readily available water contents utilized by crops are the CSW contents that make up the maximum field capacity. Moreover, when field capacity is not accessible by the plants due to the strong bonding of remaining water with soil, the permanent wilting point is achieved which is actually the point of no water uptake by the plants (Ben-Noah et al. 2021). At this point, the crop water uptake forces cannot overcome the available soil–water, which is often calculated at -15 bar. Hence, the water draw point for crops lies between field capacity and the permanent wilting point.

### Soil and water interactions affect nutrient uptake

The water storage in soil has a direct relationship with the movement of water in soil pores, making water potential and SWCs relatable (Richards 2004). The soil texture and layering profiles also influence the water flow (Khaled and Fawy 2011). The manner in which soil–water interacts with soil particles has varying effects on the absorption of water and nutrients by crops. The disparity between soil and crop root water potential serves as a determining factor in facilitating the process of crop water uptake (Vico et al. 2023).

The instantaneous water concentration and force generated by water inside the crop roots are called crop root water potential. This is the key determinant for the direction of water movement in or out of the plant, because water movement is always explained as spontaneous from higher to lower potentials (Agegnehu et al. 2016). The pressure potential, turgor pressure, and solute potentials are also the denominators for crop root water potential (Boyer 2015). However, the real factor that governs water flow is the interaction of water molecules with soil (adhesion) and with each other (cohesion). Gravitational pull is another key factor that restricts uphill water flow (Miranda-Apodaca et al. 2018).

**Fig. 1:**
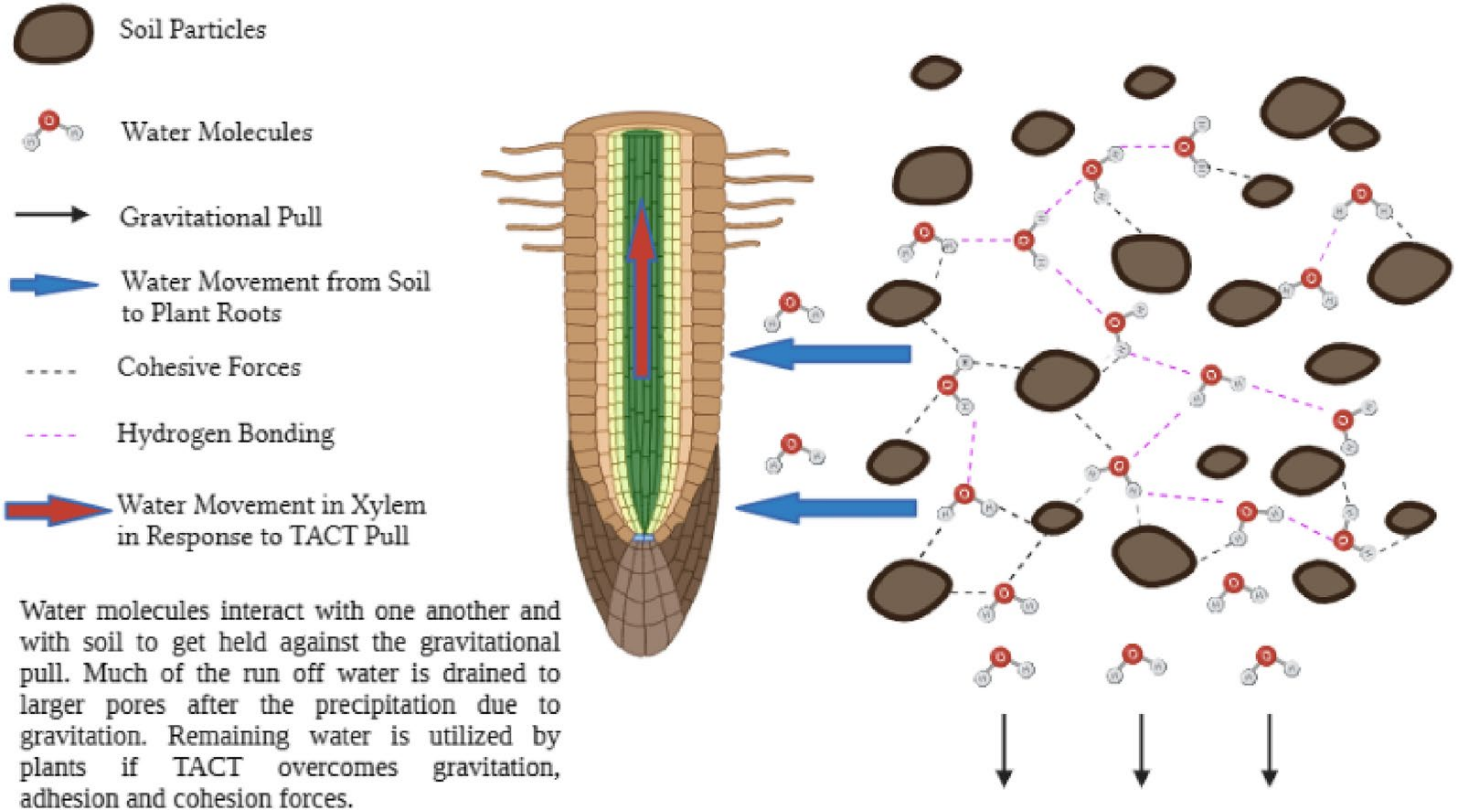
Schematics of water–water and water–soil interaction affecting the movement and availability of SWCs to plant roots (Drawn using Biorender.com)

In general, water molecules are associated with one another, and plants are only able to uptake soil–water when they overcome the adhesion and gravitational pull. Therefore, the water uptake by the plants, even at more feasible bar pressure is difficult with varying soil textures and porosity. The water movement from soil to plant roots is justified by the suction phenomena elucidated by TACT theory (transpiration, adhesion, cohesion, and tension). The present theory elucidates the mechanism underlying water transport within the xylem and the generation of negative water potential within plant roots, resulting in the development of suction forces that facilitate the uptake of water into the plant roots (Lambers et al. 2019). The supporters of this theory are of the view that water suction is only possible when the TACT forces overcome the gravitational pull and other physical interactions established by water. The schematic of TACT and water–soil interaction is explained in Fig. 1.

The disparities in nutrient composition between soil and crop roots facilitate the process by which crops absorb nutrients. The plant nutrient concentration refers to the quantity of nutrients found in the sap of a plant, which is measured as a ratio of mass or molarity per unit volume (Chen et al. 2022). Plant nutrient concentration varies with plant species, growth stage, and environmental conditions. Crop uptake of water and nutrients also depends on the characteristics of the root system, such as root branching pattern, root surface area, root diameter, root length, root hair density, root depth distribution, etc. (Dotaniya and Meena 2015). The characteristics of the root system affect the contact area between roots and soil particles as well as the transport capacity of roots (Pregitzer and King 2005).

Crops need adequate amounts of both water and nutrients for optimal growth and yield. The interaction of soil–water with soil particles depends on various factors, such as soil texture, structure, organic matter, pH, cation exchange capacity, fertilizer application, climate, crop species, growth stage, environmental conditions, root system characteristics, etc. Therefore, understanding these factors and their effects on soil–water–plant relationships is important for managing soil fertility and irrigation practices effectively (Fu et al. 2019).

The relative proportions of clay, silt, and sand are expressed as soil texture (Barman and Choudhury 2020). The texture of soils is considered to influence nutrient availability either by changing the water holding capacity or by manipulating the cation exchange capacity (Sharma et al. 2015). The water retained by soil for plant usage is subjected to water holding capacity, as this is the most crucial feature that supports nutrient uptake and transport mechanisms in plants. In general, higher water-holding capacity is a feature of finer-textured soils (clayey soils) than coarser textured soils (sandy soils), because they have 8–10 times more total pore space and smaller pores that hold water more tightly (Li et al. 2014). This means that clayey soils can provide more water and nutrients to plants than sandy soils, especially during drought periods. However, clayey soils can also become waterlogged or anaerobic if drainage is poor, which can limit nutrient availability and plant growth (Wang et al. 2021a).

The concentration of soil nutrients exhibits variability based on factors, such as soil texture, structure, organic matter content, pH levels, cation exchange capacity, and the application of fertilizers (Bouajila et al. 2023). In general, finer-textured soils (finer than 1 mm) have higher soil nutrient concentrations than coarser-textured soils, because they have a larger surface area and a more negative charge on their surfaces that can adsorb cations (positively charged nutrients). However, finer-textured soils can also bind some nutrients too strongly or make them unavailable by forming insoluble compounds with other elements.

Cation exchange capacity (CEC) reflects the availability of cationic nutrients present in soil–water, such as ammonium, calcium, potassium, magnesium, iron, zinc, etc. Cover crops in seed maize or soybean treatment (SCCC) had a significant effect on soil exchangeable K in the topsoil (0–5 cm soil layer) (Emamgolizadeh et al. 2015). These positively charged ions are found in close interaction with negatively charged organic soil constituents. The positively charged ions are absorbed by the plant root using an anti-port cation exchange mechanism (Ulusoy et al. 2016). In general, finer-textured soils have a higher CEC than coarser-textured soils, because they have a larger surface area and more negative charge on their surfaces. This means that clayey soils can store more cations and prevent their leaching than sandy soils. However, clayey soils can also bind some cations too strongly or make them unavailable by forming insoluble compounds with other elements, such as phosphorus.

Soil texture also affects the mobility and retention of negatively charged nutrients (anions), such as nitrate, phosphate, sulfate, etc. Previously, 10% clay soil addition was nearly as effective in reducing N and P leaching as 20% clay soil. Adding only 10% clay soil to a sandy soil is likely to be less expensive than 20%. (Yan et al. 2022). Anions are not held by the soil particles but move freely with the soil–water. In general, coarser-textured soils have higher anion leaching potential than finer-textured soils, because they have larger pores that allow more water flow. This means that sandy soils can lose more anions by leaching than clayey soils, especially under high rainfall or irrigation conditions (Ali et al. 2020). However, sandy soils can also allow more anion uptake by plant roots than clayey soils, because they have lower anion adsorption potential. Convincingly, soil texture affects nutrient availability by influencing the water-holding capacity and the cation exchange capacity of the soil. Finer-textured soils tend to have higher nutrient retention and lower nutrient leaching than coarser-textured soils, but they may also have lower nutrient availability and aeration under certain conditions (Liu et al. 2021). Therefore, soil texture needs to be considered when managing soil fertility and applying fertilizers.

### SWCs in different soil types

The predominant land textures in arable areas primarily depend on precipitation to sustain SWCs, which is crucial for supporting arid vegetation and facilitating the ecohydrological cycle (Xu et al. 2023). The SWCs in arid and semi-arid regions exhibit increased heterogeneity in response to variations in precipitation patterns and vegetation types (Obade and Gaya 2021). Due to the exhaustive evaporation factor, the smaller precipitation index has insignificant effects on SWC (Wilson et al. 2004). SWC is reported to decrease with increasing soil depth due to lessening influence of precipitation factors on deep soils. Such as, the coefficient of variation was found to be high for SWC in the horizontal direction (48%), but was relatively small for SWC in the vertical direction (9%) (Zhao et al. 2017). Therefore, it can be established that precipitation and SWCs are strongly associated with local climate (Mei et al. 2019). Diverse microbial communities coexist in various soil types to maintain their textural heterogeneity at microscale (Huang et al. 2023). Soil textures mainly hinge on soil heterogeneity, which has a direct linkage to soil pores distribution (Rooney et al. 2022). The differential pore distribution affects the SWC and pore saturation at large; therefore, soil–microbial flora can also influence the SWC measurement techniques that involve EM waves. The development of more microbial communities in soil pores often uplifts the water density (Amarasekare 2003) and also adds to the relative water volume, which later on results in false positivity for volumetric SWC observations (Vos et al. 2013). Scientists believe that this microbial flora is involved in the natural biogeochemical cycles and offers colonization resistance to the soil (Stein et al. 2014).

The water matrix suction by the crops has been a recent topic of research in environmental science and agriculture engineering (Xu and Yang 2018; Rahardjo et al. 2019; Tian et al. 2020). Measurement of SWCs in specific soil types is limited to detection techniques whether recent or advanced (Wang et al. 2021; Karakan 2022; Ojeda Olivares et al. 2020). These studies have presented the idea of hydraulic retention and SWC strength using quantitative analytical techniques. Soil texture (Liu et al. 2012) and pore conformations (Chen et al. 2017) have been found to pose a significant influence on total SWCs. Moreover, the initial soil wetting has also been investigated to impact the water content at large (Zhang et al. 2021). However, the rapid draining of water due to gravitational pull generates a sufficient number of atrocities in data collection and further analytical processing.

**Fig. 2:**
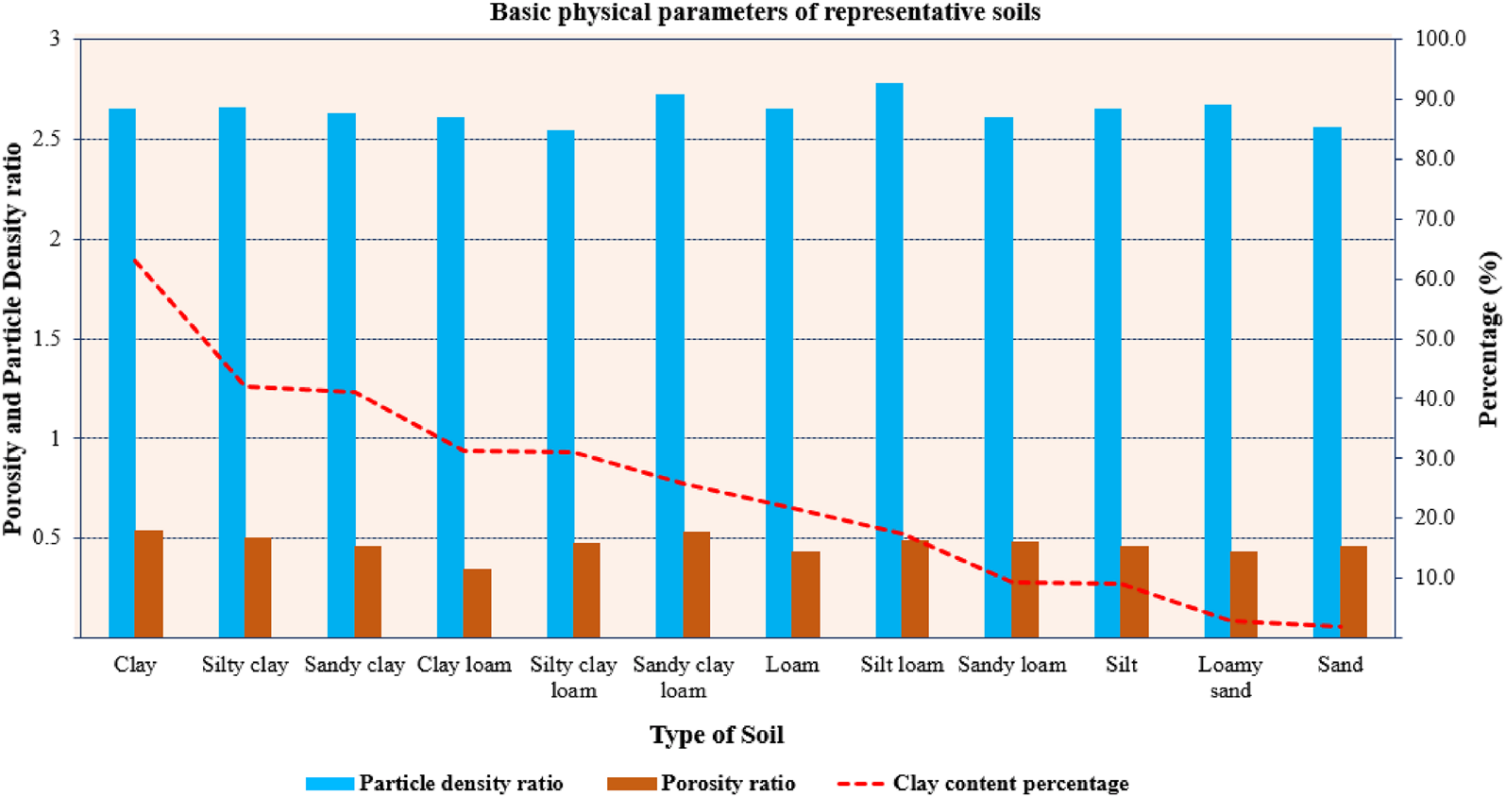
Physical parameters of differently textured soils in relation to water contents (Redrawn from data source) open access license (Obade and Gaya 2021)

**Fig. 3:**
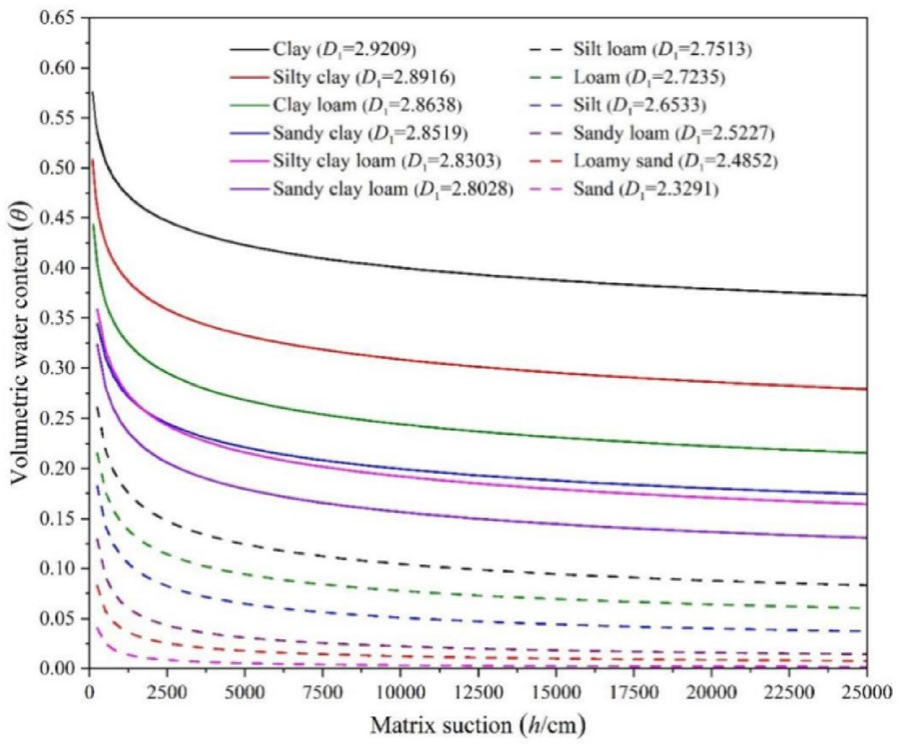
Results of fractal model showing the association between VSWC and matrix suction from diverse soil textures (Reprint from open access license) (Obade and Gaya 2021)

In another study, it was found that out of 120 samples, the water contents of more fine clayey soils were significantly higher compared with those of more sandy soils (Li et al. 2016). Nonetheless, the finer and clayey soil holds water sufficiently well and halts water mobility for crops even at ideal water bar of -30 units. Furthermore, when the soils are tuned to be finer, the porosity may increase, but the crop efficacy to drag water from these pores is significantly reduced (Fig. 2). Moreover, the suction matrix fractal analysis model for VSWC in various soil types confirms the significantly differential VSWCs (Fig. 3).

In general, sandy soils have higher hydraulic conductivity than clayey soils, because they have larger pores that offer less resistance to water flow (Hao et al. 2019). This means that sandy soils can drain faster than clayey soils after rainfall or irrigation. However, this also means that sandy soils can lose more water by evaporation or transpiration than clayey soils, because they have a lower matric potential and cannot retain water against atmospheric demand. Because clayey soils have smaller pores that give more barrier to water movement, they have poorer hydraulic conductivity than sandy soils. This means that clayey soils can hold more water after rainfall or irrigation than sandy soils. However, because clayey soils have a larger matric potential, which prevents gravitational drainage, they can become waterlogged or anaerobic if drainage is poor (Mulla et al. 2023). SWCs vary with soil texture due to differences in pore space and pore size distribution. Clayey soils have a higher SWC and lower hydraulic conductivity than sandy soils under all moisture conditions. This affects the water holding capacity and water movement in the soil, which in turn affect various processes, such as plant growth, nutrient cycling, water balance, and soil erosion. Therefore, understanding the relationship between SWC and soil texture is important for managing soil and water resources effectively.

### Advancements in SWCs measurements

Soil–water content (SWC) is a crucial parameter that affects various biophysical processes, such as plant growth, nutrient cycling, and water balance (Pereira et al. 2020). Measuring SWC accurately and efficiently is important for many applications in ecology, agriculture, hydrology, and engineering. The SWC is often termed wetness of soil, which in general has no specified but relative value (Lal and Shukla 2004). This relative value is often a ratio, often calculated as water over soil volume (V_w_/V_S_) and volumetric SWC (VSWC), is usually represented as *θ* (García-Gamero et al. 2022*).* Moreover, as a general convention, this VSWC is often referred to as relative soil volume filled with water. Being a basic quantity for soil research initiation, SWC measurement is a routine analysis for soil examination. There are diverse methods that have been reported to measure SWC and have their own pros and cons. One of the basic, or so-called absolute, methods of measuring SWC is the gravimetric method (GM), which is noncalibrated and the most basic method.

The GM is a destructive approach that often fails to provide real-time knowledge and cannot measure the same sample area again (Villalobos et al. 2008). This method may also leave a space from where the sample has been taken, which will abruptly change the SWC, bulk density, and relative volume of nearby areas. These factors might leave the GM method unreliable and nonrepresentative if we are more involved in the real-time measurement of SWC. The neutron method (NM) was then developed for more likely real-time and non-destructive SWC measurements. This method accounts for only elastic collisions between neutrons and water molecules. Consequently, the presence of tightly bound hygroscopic water molecules and neutron dissipation can lead to the acquisition of false-positive outcomes.

The determination of the actual water content accessible for crops may continue to lack definitive findings (Drizo et al. 2022). Due to high radiation levels, this method also remains banned in most countries. This method is non-destructive, continuous, and capable of measuring SWC at different depths and large volumes of soil. However, it is expensive, hazardous, and requires a license to operate. It also requires calibration with other methods and correction for soil bulk density and temperature. With the research and development of SWC measurement technology, more accurate and noninvasive methods were also introduced, which require little calibration and are more sensitive. These methods either rely on electrical or electromagnetic (EM) signal travel in the soil and water, and then the resistance, capacitance, frequency, or time of travel can be compared as a measure of SWC. These methods are more robust and require prior installation and calibration (Karimi, et al. 2020). However, once installed, real-time and noise-free results can be obtained.

It remains a key factor that electromagnetic wave propagation is inhibited by air gaps, so the techniques utilizing EM waves are limited to certain soil types. For instance, the ground penetration radar (GPR) method involves EM wave propagation in soil and then a reflection of these waves by soil entities. It uses a transmitter and a receiver that are moved along the soil surface or placed in boreholes and measure the travel time, amplitude, frequency, or envelope of the reflected waves (Pandya 2021). This method is non-destructive, high-resolution, and capable of measuring SWC at different depths and large areas of soil. However, it is complex, expensive, and affected by soil texture, structure, salinity, and surface roughness. It also requires inversion models and calibration with other methods (Sharma and Sen 2022). The comparative analysis of the techniques discussed is also summarized in Table 1.

**Table 1:** Comparison of advancements in SWCs measurements

Technique	Principle	Advantage	Disadvantage	References
Gravimetric method (GM)	Soil collection and analysis	✓ Absolute results	✓ Non-calibrated ✓ Destructive	Lal and Shukla (2004)
Neutron method (NM)	High energy neutrons	✓ Real time ✓ Non-destructive	✓ High radiation ✓ Considers only elastic collisions	García-Gamero et al. (2022)
Ground penetrating radar (GPR)	Electro-magnetic Waves	✓ Non-destructive High resolution	✓ Complex ✓ Expensive ✓ Requires calibration	Karimi et al. (2020)
Capacitance method (CM)	Resistance and capacitance sensors	✓ Relatively inexpensive ✓ Automated	✓ Difficult installation ✓ Results affected by soil types	Bünemann et al. (2018)

Conclusively, there are various techniques available for measuring SWC at different scales and for different purposes. Each technique has its own strengths and weaknesses that need to be considered when choosing the most suitable one for a given situation. There is no single best technique that can measure SWC universally and accurately. Therefore, it is often necessary to combine or compare different techniques to obtain reliable and representative estimates of SWC. Future development of SWC measurement techniques may focus on improving their accuracy, precision, resolution, cost-effectiveness, ease of use, and integration with other sensors, models, and programs developed by AI.

### Soil texture prediction and analysis using artificial intelligence

Soil texture has been found to play a crucial role in ecosystem health, agricultural production, and sustainable farmland management (Zhai et al. 2006). Among the diverse soil properties, the texture plays a pivotal role in decision-making for the planning and management of agricultural land. The conventional approaches with agriculture sensors and statistical analysis were found to be non-robust, time-consuming, non-instantaneous, and expensive (Bormann 2010). However, with advanced AI processing tools and ML applications, new avenues for texture prediction and revolutionized soil management practices have been opened.

Conventional soil texture analysis is performed by sieving, sedimentation, and other hydrometric laboratory methods. Later, the results from these experiments are statistically analyzed, and conclusions are drawn manually. The complexity of this analysis can be presumed from the variable soil textures and environmental attributes that affect it. This creates heaps of data that cannot be translated into a single conclusion for correct decision-making (Riese and Keller 2019). Therefore, all of these manual dealings require skilled professionals, a significant amount of time, and specialized instruments. However, AI tools are a promising set of alternatives for these limitations that otherwise confine soil management.

AI techniques that include machine learning (ML) and deep learning (DL) are potentially remarkable for accurate and efficient soil texture predictions. The inputs utilized by these algorithms are compositional, spectral, and geographical data sets that can be in non-numerical form (Johnson et al. 2020). AI processing of these data sets mainly reduces the cost, time, and labor involved compared with conventional laboratory protocols. The complexity of relationships among the data sets is quickly learned and applied using the ML and DL algorithms (Wang et al. 2021b). This is not the only scale available with this technology; cloud systems and mobile applications are another step forward. The wider scalability of AI enables farmers and land managers to access and process land management operations with ease.

The subjective and error-prone data analysis from traditionally practiced laboratory protocols is then dazzled by the objective, more accurate, and real-time data processing using AI tools (Hassan-Esfahani et al. 2015). By leveraging AI techniques, we can overcome the limitations of traditional laboratory-based methods and enable real-time decision-making in soil management practices. However, addressing challenges related to data quality, interpretability, and system integration will be crucial for the successful implementation of AI-based soil texture analysis (Liu et al. 2022). With continued research, collaboration, and innovation, AI-driven soil analysis can contribute significantly to sustainable land management, agricultural productivity, and environmental conservation. Integrating AI models seamlessly into existing soil management practices and decision support systems requires collaboration among scientists, engineers, and policymakers. This integration would ensure the practical implementation of AI-based soil texture analysis on a broader scale.

### Artificial intelligence and SWC measurement

**Fig. 4:**
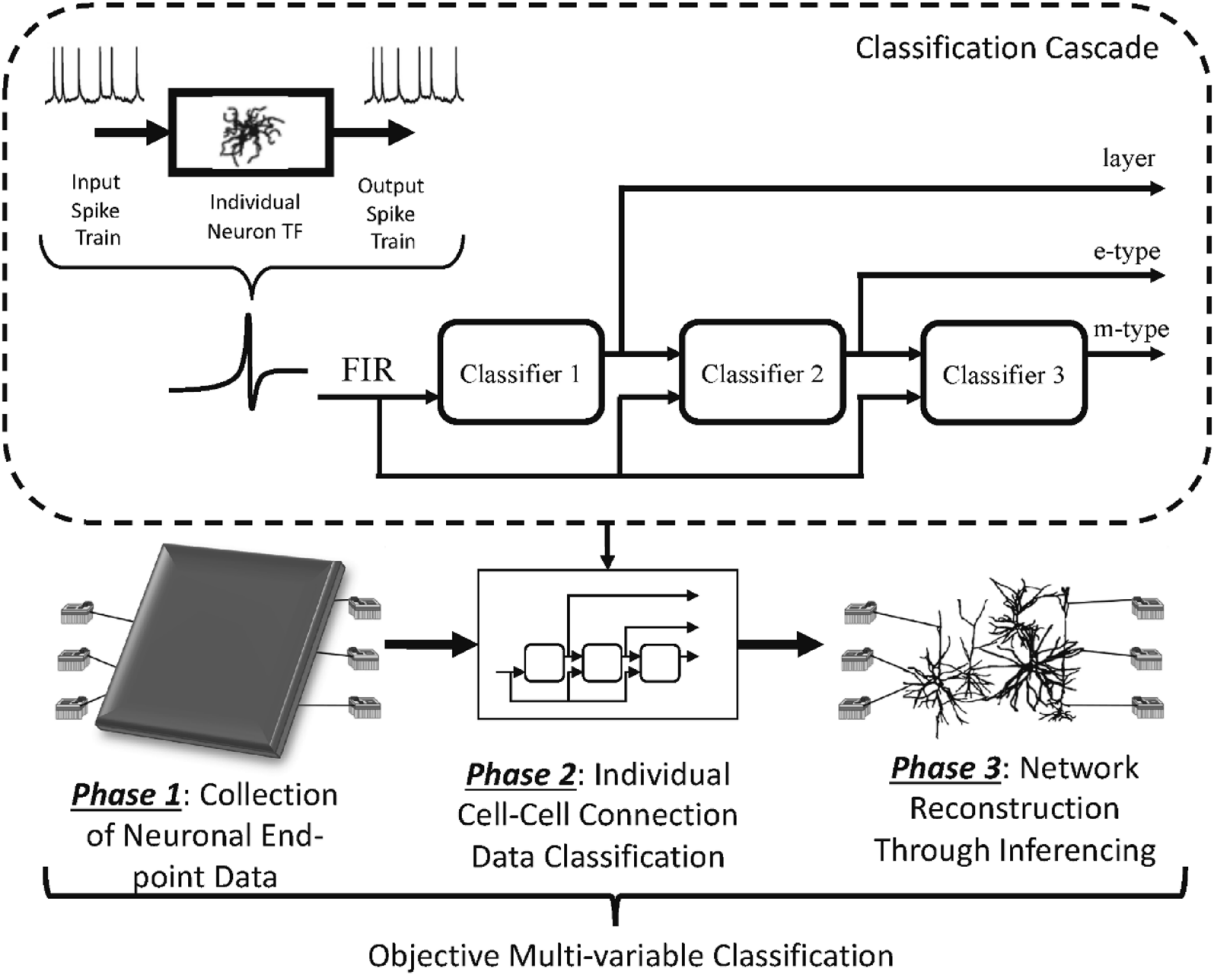
Comparison of artificial and biological neuron (Barros et al. 2022) (Open Access)

The highest cadre of the information technology revolution is AI, which has influenced and reshaped every field of life. AI has a robust approach, where the computers learn from already existing data sets and get themselves trained enough to solve complex problems. This AI resides on very complex modules that are cladistical in their linkage and are very complex to understand (Barros et al. 2022). AI is generally compared with human neurons for its signal-processing complexity. The inputs, processing, decision-making, and outputs are similar to those of human neurological systems (Fig. 4).

A variety of fields have benefited from AI, for instance, robotics, medical imaging, disease detection, and flight control systems. AI has also been found to be capable of solving key issues in agronomy, meteorology, and hydrology. The science of SWC measurement has also benefited from AI source codes recently. It has presented itself as a water- and soil-state manager with high performance, correlation, and statistical correctness (Gao et al. 2022). This is a very smart add-on to robust agricultural decision-making under the influence of various environmental factors. This section of the manuscript will review AI strategies for agriculture biosensing, with a special focus on SWC management.

Climate variability can readily impact the outcomes of the FDR and TDR techniques for measuring SWC. This has inspired scientists to report a novel method in 2020 (Mallet et al. 2020), which included measuring the temperature response and heating the surrounding soil in short bursts. Due to its high degree of automation and being less influenced by climate, this actively heated optical fiber (AHOF) method can be applied, where conventional TDR and FDR might not be enough (Ciocca et al. 2012). However, the analysis performed by AHOF still requires correction due to unpredicted errors, types of soil, and other climatic variables. Therefore, the artificial neural network (ANN) utilized for removing the errors generated by the AHOF method to improve the effectiveness of acquired results (Liu et al. 2023). The prescribed model was recommended for usage, which contained the use of a climate layer or cover layer that was found to be highly correlated with SWC contents.

The field of agriculture sensing has now been closely linked with ANN models to rectify and filter the most accurate results and forecast the future for irrigation and agricultural management. Precision agriculture has now been supplemented with pH, humidity, SWC, mechanical, and airflow sensors that provide enough results for robust decision-making using ANN tools. Considerable efforts have been made to forecast the future of wheat crops using ANN model training based on previous sensor readings (Roshan et al. 2022). The scientists then concluded multilayered perceptron model was the most effective, with lower MSE and RMSE when compared with other ANN models. Accurate assessment of SWCs has grabbed the attention of researchers over the past few years.

Many efforts have been made to contribute to *in-situ* data collection and the development of remote databases for SWCs (Owe et al. 2008). However, these efforts are still in progress, and to date, we do not have such a database on a global scale. SWC measurement using pseudo-transfer functions (PTFs) is a solution to maximize the right soil data acquisition from lands, where there are no data available. Regression model analysis usually generates PTF data that can be fed to an adaptive neuro-fuzzy interference system (ANFIS) that gives a non-explicit insight into SWC variables (Liu et al. 2020). The research found the ANFIS model to be more efficient, robust, and quick compared to conventional statistical models that have higher standard errors (Hosseini et al. 2021). Understanding soil texture and water content is key to proper crop management. Precipitation and droughts considerably account for more than anything else that can affect soil textures (Keller and Håkansson 2010).

**Fig. 5:**
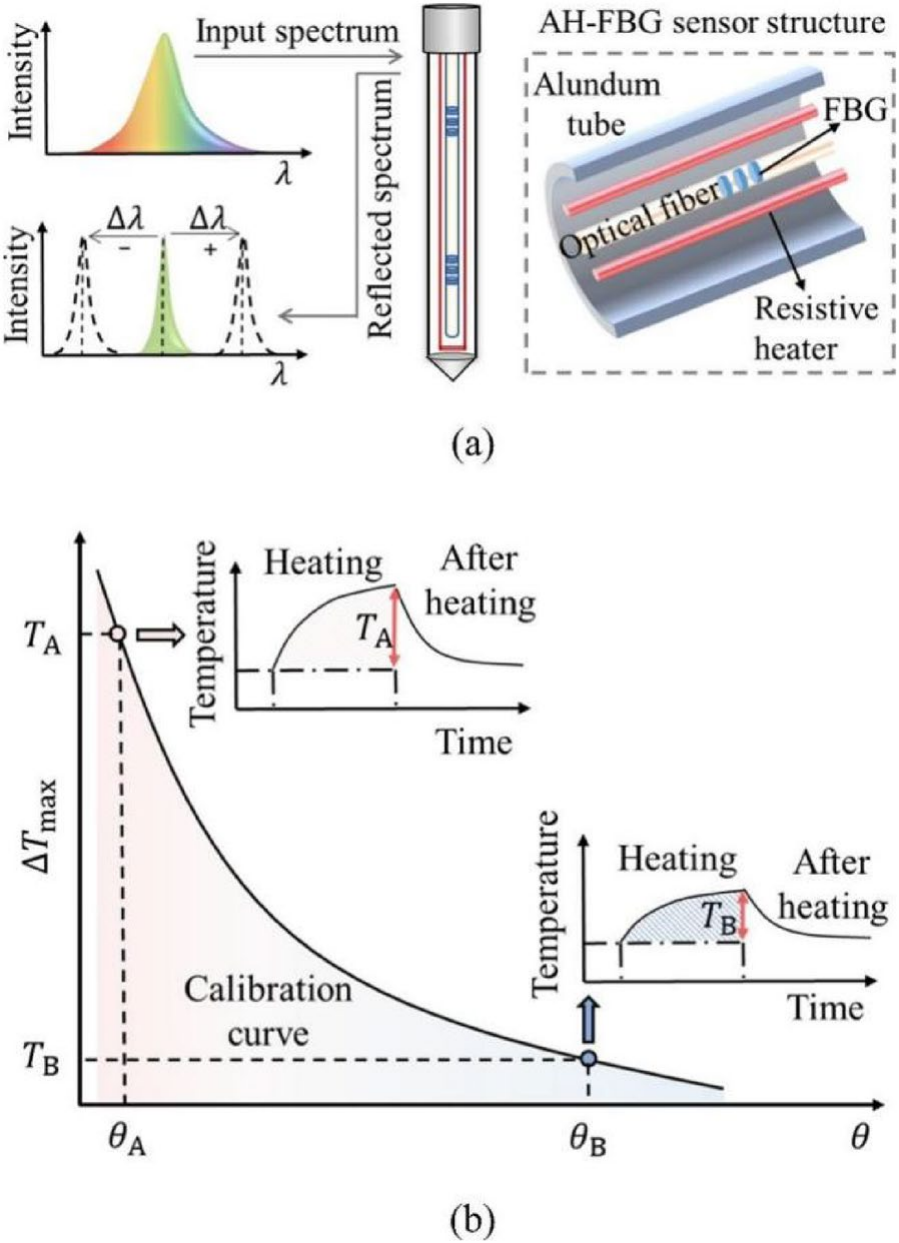
Artificial intelligence-based fiber optic sensing for soil moisture measurement (Liu et al. 2023) (**a**) an optical fiber with quasi-distributed FBG for temperature measurement (**b**) example of result plot from the fiber with quasi-distributed FBG

The main components of a Bragg grating AH–FBG moisture sensor are a resistance wire, an optical fiber with quasi-distributed FBGs for temperature monitoring, and an enclosed tube. The capacity of an FBG to reflect light waves with a specific center wavelength is affected by temperature and strain. The AH–FBG sensor's center wavelength shift is unaffected by strain, because a corundum tube encircles it, taking the strain out of the equation. If the sensor is placed in the ground, the soil temperature at the relevant measurement point may be calculated by examining the FBG reflection spectrum (Fig. 5). This superior technology, however, is strictly confined to certain situations. For example, when heated, the AH–FBG sensor may be seen as an endless linear heat source. Furthermore, the soil being tested is expected to be homogenous and isotropic (Liu et al. 2023).

### Role of AI in smart agricultural irrigation systems

AI has enabled computer science to create artificial systems that work similar to the human brain. These artificial systems or machines can help humans perform more precise learning, logical reasoning, problem-solving, and decision-making. Due to its enormous potential, AI has been integrated into many research fields, such as agriculture, where it can help farmers optimize their use of water, land, and other resources, increase their productivity and profitability, and reduce their environmental impact (Kose et al. 2022). Agriculture faces the key challenge of water scarcity in regions with low precipitation and where droughts are prevalent.

The Food and Agriculture Organization (FAO) states that agriculture accounts for about 70% of global freshwater withdrawals, and it can go up to 15% by 2050 to meet the mounting demand for food by a growing population (Pernet and Ribi Forclaz 2019). However, water availability is unevenly distributed across the world, and climate change is expected to exacerbate the variability and impulsiveness of rainfall patterns, distressing crop yields and quality. To address this challenge, many researchers and practitioners have proposed and implemented smart irrigation systems that use sensors, controllers, actuators, communication networks, and data analysis tools to monitor and control the delivery of water to crops according to their needs and environmental conditions (Jong et al. 2021).

These systems aim to improve water use efficiency, reduce water waste and runoff, enhance crop growth and quality, and save energy and labor costs (Penghui et al. 2020). However, smart irrigation systems face some limitations, such as high maintenance cost, a lack of interoperability and standardization among different devices and platforms, the complexity of data processing and interpretation, and the uncertainty and variability of crop responses to irrigation. Moreover, traditional irrigation scheduling methods based on fixed rules or thresholds may not be intelligent enough to detach the dynamic and nonlinear interactions between soil, crops, water, management practices, and weather (Kouadio et al. 2018). Therefore, AI is considered to play a significant role in enhancing of functionality and performance of smart irrigation systems. Agriculture, when factionalized with AI systems, can help farmers identify instantaneous and accurate measurements as well as decisions for fertilization, irrigation, SWC, pH, and related factors (Raja and Shukla 2021). AI can also help farmers optimize their irrigation schedules and distribution using machine learning algorithms that can learn from historical and current data, forecast future scenarios, and adapt to changing conditions.

There are a variety of insights available, where AI can help smart irrigation systems obtaining optimal results. Fuzzy logic is one of the examples that deals with imprecise and uncertain information using linguistic variables and rules instead of numerical values. Fuzzy logic can be used to model complex systems, such as SWC, soil–total moisture, nutrient dynamics, or crop water requirements by incorporating expert knowledge and human intuition (Mahmoudi et al. 2022). Second, the Internet of Things (IoT) is a network of interconnected devices that can collect, transmit, process, and act on data without human intervention. IoT can be used to implement smart irrigation systems by integrating sensors, controllers, actuators, communication modules, cloud computing services, and mobile applications (Kodali and Sahu 2016).

Finally, ML is another smart advancement that is empowered by ANNs, DL, and reinforcement learning (RL). These attributes of ML have the advantage of processing soil imagery to extract information about soil textures, SWCs, nutrition, and other parameters (Pham et al. 2018). Therefore, utilizing these resources efficiently over conventional statistical approaches and vigorous data analysis can be the new era approach for smarter agriculture practices, especially for soil management. In the last couple of years, numerous researchers and engineers worked on different types of materials subject to biological sciences, agricultural sciences and environments.

### Statistical and AI tools comparison

The statistical approaches have served the science from its beginning and have presented enormous ways of data analysis for further decision-making and drawing conclusions (Phoon et al. 2010a). Most mathematical or statistical approaches involve numerical data processing, stigmatization, and drawing data correlations. Rather, the AI tools rely on computational modules to carry out such challenging tasks that otherwise require human intelligence. Drawing the line between statistical analysis and AI processing is difficult due to the reliability of both in various conditions (Henderson et al. 1992). However, AI processing for the same subject of experiments can be more advantageous, robust, and decisive compared to statistics. Statistical analysis usually requires structured and large numerical data inputs, such as SWCs, soil properties, weather data, etc. (Phoon et al. 2010b). However, on the other hand, AI tools can efficiently operate with non-structured and qualitative data, such as images, videos, texts, voice data, etc. (Yu and Kumbier 2018). In addition, the AI tools are smart enough to handle data augmentation, regularization, and ensemble learning of small and noisy data sets (Friedrich et al. 2022). More specifically, ML tools are data-driven models and novel algorithms for training, processing, and making predictions for given data sets that are not addressed while using predefined statistical models. The interpretation and visualization of data have been simplified for human interface in AI tools, but the statistical approach is not robust and time-consuming for this purpose. Statistical tools can be used for descriptive and inferential purposes, such as describing the distribution of soil–water content, identifying the factors that affect soil–water content, testing the differences or relationships among soil–water content variables (Wadoux et al. 2020). On the other side, AI tools can be used for predictive and prescriptive purposes, such as predicting SWCs based on various inputs, optimizing the irrigation schedule based on SWC goals, recommending the best management practices, etc. Statistical and AI tools are known to have different strengths and limitations for SWC analysis. Depending on the research question and objective, one may choose to use either or both of them to obtain comprehensive and accurate insights from the data. The link between statistical and AI tools is an advanced key to opening the doors for robust management and agricultural biosensing (Table 2).

**Table 2:** Comparison of data processing with AI tools and statistical tools

Advantage	AI processing of data	Statistical tool
Handling complex data	Structured and unstructured text, images, etc.	Designed for structured data with known distributions
Predictive power	Forecasting, classification, and anomaly detection are possible	Primarily focused on hypothesis testing and descriptive statistics; may not excel in predictive tasks
Automation of task	Can automate data preprocessing	Requires more manual intervention
Adaptability	Can adapt to changing patterns and learn from data	Unadaptable and may need manual adjustments
Real-time decision-making	Enables real-time decision-making by deploying models as APIs or microservices, supporting applications and services	Used for batch processing and may need manual integration for real-time decision support

### Development of a global dataset for soil texture and SWC data

Many ecological processes, water availability, and agricultural productivity depend on the type and strength of agricultural land. Soil texture and SWC are the more crucial parameters to determine effective land usage, sustainable agriculture, and water administration (Maino et al. 2022). However, due to the vast earth’s surface and spatial soil heterogeneity, it is very difficult to obtain comprehensive and reliable soil information (Martinelli and Gasser 2022). The global data set of SWC and soil texture information has the potential to enable farmers, policymakers, and land managers to timely engage in smart agricultural practices. Such data sets can also help in crucial hydrological analysis and climate modeling.

Soil–water dynamics, climate change, flood prediction, and water movement modeling can be reshaped after the development of these global data sets. Environmental conservation is another crucial concern that can be smartly managed after developing global data sets for soil parameters (Zhang and Shi 2019). This will help in the assessment of soil erosion, fertility, and ecological health. Moreover, areas with vulnerable soils can be identified more accurately, and targeted conservation and land restoration efforts can be practiced more efficiently. Considerable efforts have been made in past toward the development of a global soil parameter database. These efforts involve everything from general laboratory analysis of soil from various localities to the installation of advanced sensors globally or geospatial technologies (Mallah et al. 2022).

Remote sensing technologies, such as satellite imagery and airborne sensors, can provide valuable information on soil properties indirectly. Spectral signatures obtained from these sensors can be correlated with soil texture and soil–water content data collected from ground-based measurements. Proximal sensing techniques, such as electromagnetic induction and ground-penetrating radar, also contribute to the acquisition of soil data on a larger scale. Machine learning algorithms, such as random forests, support vector machines, and neural networks, can be employed to develop predictive models based on available soil data and auxiliary environmental variables. Geostatistical techniques, including kriging and co-kriging, help interpolate and extrapolate soil property values to unsampled locations, improving the spatial representation of the dataset (Naimi et al. 2022).

Ensuring an adequate distribution of soil samples across different regions, soil types, and land cover categories is essential for capturing the spatial heterogeneity of soils globally. Sampling biases and limited access to certain regions can pose challenges in achieving a representative data set. Soil data collected using different protocols, laboratory methods, and instruments needs to be standardized and harmonized to ensure consistency and compatibility. Developing robust quality control procedures and data harmonization protocols is necessary to integrate diverse data sets into a coherent global database. Encouraging data sharing among researchers, institutions, and national soil agencies is crucial for developing a comprehensive global data set. Collaboration at regional and international levels can help overcome data gaps and promote data exchange, leading to a completer and more reliable dataset.

## Conclusion

The need for robust, quick, and accurate soil analysis using AI technology holds a great and promising future for sustainable agricultural practices and efficient natural resource management (Pandey et al. 2023). The nonuniform geospatial distribution of SWCs and soil textures is a big impediment to the development of a global soil database. Even advanced statistical data processing is time-consuming and has delayed decision-making to practice intelligent agriculture. However, by leveraging AI, DL, and ML techniques, researchers can overcome these challenges and obtain more efficient and reliable soil analysis results. Artificial neural networks (ANNs) and other related AI modules have shown promising results in achieving robustness in SWC and soil texture analysis. These techniques allow for the processing of non-numeric geospatial data, providing valuable insights for soil scientists and aiding in the development of a global SWC database.

Machine learning algorithms, such as random forests, support vector machines, and neural networks, can be employed to develop predictive models based on available soil data and auxiliary environmental variables. In addition, geostatistical techniques such as kriging and co-kriging play a significant role in interpolating and extrapolating soil property values, improving the spatial representation of the data set. However, challenges such as false positivity in SWC results and bugs in advanced detection techniques need to be addressed to ensure accurate and reliable soil analysis. Further research and development are required to refine AI models and improve their performance in soil analysis applications. It is evident that conventional statistical tools alone are insufficient for robust SWC and soil texture analysis. The integration of AI and related technologies provides a promising pathway to enhance soil analysis efficiency, enable intelligent decision-making, and facilitate sustainable land management practices. By harnessing the power of AI, researchers can make significant strides in understanding soil–water relationships, improving agricultural productivity, and developing a comprehensive global SWC database to support sustainable agriculture and resource management.

## Data Availability

Data sharing not applicable to this article as no data sets were generated or analyzed during the current study.
